# “Passing through difficult times”: Perceptions of perinatal depression and treatment needs in Malawi - A qualitative study to inform the development of a culturally sensitive intervention

**DOI:** 10.1371/journal.pone.0217102

**Published:** 2019-06-18

**Authors:** Mwawi Ng’oma, Samantha Meltzer-Brody, Ellen Chirwa, Robert C. Stewart

**Affiliations:** 1 Department of Mental Health, University of Malawi College of Medicine, Blantyre, Malawi; 2 St John of God Hospitaller Services, Malawi, Lilongwe, Malawi; 3 Department of Psychiatry, University of North Carolina at Chapel Hill, Chapel Hill, North Carolina, United States of America; 4 Faculty of Midwifery, University of Malawi Kamuzu College of Nursing, Blantyre, Malawi; Medical Research Council, SOUTH AFRICA

## Abstract

**Purpose:**

This study was conducted to explore the perceptions of perinatal women and key maternal care health workers about perinatal depression and the health service needs required to inform development of a culturally sensitive and acceptable psychosocial intervention.

**Methods:**

This qualitative study used a descriptive exploratory design; it is the first phase of a larger mixed methods study aimed at adapting a psychosocial intervention for perinatal depression. We conducted in-depth interviews with 22 women who screened positive for depression using a locally validated Chichewa version of the Edinburgh Postnatal Depression Scale at antenatal and postnatal clinics in 1 rural and 1 urban health care setting in Lilongwe District, Malawi. We also conducted 10 key informant interviews with maternal care health workers. Informed consent was obtained from all participants. An interview guide was used to guide enquiry about perceptions of perinatal depression and health service needs. Interviews were transcribed, translated and analysed using content analysis approach.

**Results:**

Perinatal depression was recognized as a common mental health problem that affected self-care activities and functioning of women in the perinatal period. Financial difficulties, relationship problems (polygamy, lack of support, neglect, and infidelity), traumatic events (intimate partner violence and loss) and fear of birth outcomes were identified as causes of depression. All study participants acknowledged the need for support and an intervention that will address the identified challenges. Additionally, they viewed strengthening the health delivery system as crucial to effectively address their needs and gaps identified in the system.

**Conclusion:**

The results of this study support plans to develop a family focused intervention for perinatal depression in Malawi addressing relationship, psychosocial and economic issues. It also highlights the importance of strengthening the health delivery system especially at primary care level where the majority of women access care in Malawi and across Sub-Saharan Africa.

## Introduction

Perinatal depression, defined as depression occurring either during pregnancy or in the year following childbirth [1], is a major public health problem for women. Approximately 11.9% of women suffer from perinatal depression globally [2] and the prevalence of perinatal depression is higher in women from Low and Middle Income Countries (LMIC). An analysis of five low and middle-income countries (Ethiopia, India, Nepal, South Africa and Uganda) found the prevalence of perinatal depression ranged from 3–50% [3]. In Sub-Saharan Africa, results of individual antenatal and postnatal depression studies have varied widely[4–7], with weighted prevalence rates of 11.3% for antenatal depression and 18.3% for postpartum depression reported in a systematic review of African studies [8]. In Malawi, Stewart et al [9] reported a prevalence of 10.7% for antenatal major depression and 13.9% amongst mothers of young children attending a child health clinic [10].

Risk factors for perinatal depression faced by women in sub-Saharan Africa, include: HIV/AIDS [10–12], loss of a child or loved one [13], low social support [9,14], poverty [10,13–16], intimate partner violence [9,14,17,18] and relationship problems [10,13]. Perinatal depression has also been associated with unplanned pregnancy, a previous history of depression or anxiety, food insecurity and past experience of life threatening events [4,5,7,9,19]. Perinatal depression has long term consequences: depression in pregnancy diminishes a woman’s capacity for self-care, which further compromises a woman’s physical and mental health. Infants and children are particularly vulnerable because of impaired maternal-infant interactions [20]. Further, antenatal depression is associated with adverse foetal growth and has negative impacts on child nutrition and development [20–24]. However, despite the high prevalence of perinatal depression and its consequences, and the growing evidence of effective psychosocial interventions in LMIC such as the WHO-endorsed “Thinking Healthy Programme”[25,26], perinatal women in Malawi do not currently receive any routine screening or intervention for depression during this period. The focus of reproductive health services is solely on the physical health of the mother and the infant/child leading to neglect of mental health concerns.

Previous qualitative studies conducted in Sub-Saharan Africa including Malawi reported that depression in the perinatal period was related to stressful life events [27–29] and the internal conflicts women in this region face when trying to assert themselves [30]. These studies further revealed unmet health care needs and barriers to accessing care [31], which are important for informing development of treatment programmes.

There has been no qualitative study in Malawi that has assessed the views of depressed women and maternal health care workers about the experience of perinatal depression and treatment interventions. To address this gap in knowledge, we conducted a qualitative study to explore experiences of perinatal women with depression, the challenges they meet; their coping strategies; and their service needs. The study also explored perceptions of key maternal care health workers regarding perinatal depression and a potential intervention that could be implemented in a community setting.

## Materials and methods

This study is the first phase of a larger mixed methods study aimed at adapting a psychosocial intervention for perinatal depression. We used a qualitative method, exploratory descriptive design [32] to explore perceptions of perinatal women and key maternal care health workers on perinatal depression and service needs.

### Setting

This study was conducted in two sites within Lilongwe District in Malawi: Area 25 Primary Health Care (PHC) clinic and Kabudula rural hospital. These sites were selected because they serve large populations and are representative of urban and rural settings. Data from these settings can be transferable to other PHC care settings in Malawi. Lilongwe district has a population of 2,626,901 [33]. The general fertility rate is 158 per 1000 of those women between the ages of 15–44 [34]. Notably, 12% of women have never attained any education and 62% have completed primary school [35]. HIV prevalence is 9.5% among women and 5.5% among men [35]. According to the District Social Economic Profile (SEP), population pressure and issues of climate change affect people’s livelihoods and hinder economic growth in the district. Approximately 76% of residents live in sub-standard housing and/or informal settlements. These areas are characterized by lack of access to public services, tenure insecurity, and inadequate housing. A quarter of the residents are also officially estimated to live below the poverty line, with 9% considered ultra-poor [36]. The district has 536,220 households, which are in 9191 villages under 18 Traditional Authorities [37].

Area 25 PHC clinic and Kabudula rural hospital are under Lilongwe District Health Office; they provide antenatal, delivery and postnatal services, under five child health services and general outpatient health services. Area 25 health center is located in a commercial/trading high density area in the Northwest part of Lilongwe City, an urban location with both formal and informal settlements, with 152 villages, a population of 59,150, and 13,604 women of child bearing age [37]. Kabudula rural hospital is about 65 km away from Lilongwe District Health Office in the north west of Lilongwe, a rural location, with 124 villages, a population of 41, 421, and 9, 525 women of child bearing age [37]. The main economic activity in Kabudula is subsistence farming.

Ninety seven percent (97%) of women in Central Malawi (where Lilongwe District is located), access antenatal care from a skilled provider (Doctor, Clinical Officer, Medical Assistant, Nurse or Midwife) and 76% of children receive all basic vaccinations while 71% are fully immunized [34].

### Participants’ recruitment

The nurses in charge of antenatal and postnatal clinics in the two study sites provided information about the study to perinatal women in antenatal and postnatal waiting rooms during their routine health talk sessions and directed those who showed interest to the research team. The research team thoroughly explained the study and read the information sheet to the prospective participants including the benefits and risks of the study. Those willing and who met the study inclusion criteria signed a consent form (signature or thumb print verified by a witness) and were enrolled in the study.

We purposively selected pregnant women and mothers aged 18 years and above with six weeks old babies, (n = 101 from Kabudula and n = 88 from Area 25 PHC clinic). There were 189 perinatal women screened for depression using an interviewer-delivered Chichewa version of the Edinburgh Postnatal Depression Scale [EPDS], with visual prompt cards to facilitate responses [38]. The EPDS has been used previously in Malawi following a process of validation which involved translation, back translation and testing in a population of pregnant women in a rural clinic in Malawi [38]. The criterion validation showed that the Chichewa version of the EPDS had satisfactory test characteristics as a screening measure for antenatal depression against Structured Clinical Interviews for DSM-IV (SCID) as a gold standard [38]. Out of the 189 participants screened, 37 women screened positive for depression (these had a score of 12 and above on the EPDS and/or endorsed suicidal thoughts (item 10) with or without attempt regardless of their EPDS score). These women were invited for in-depth interview. Fifteen of the 37 women were not interviewed for the following reasons: Five perinatal women were referred for diagnostic assessment and management urgently because of the severe nature of their problem, four perinatal women who had already waited a long time for their postnatal checks in the postnatal clinic refused to spend an extra hour for in-depth interviews following screening, and six postnatal women did not turn up for interviews.

Purposive sampling was also used to select key maternal care health workers. We selected six Primary Health Care workers, working in maternal and child health section (antenatal and postnatal clinics) for over a year, three from Kabudula Rural Hospital and three from Area 25 PHC Clinic. We also selected four maternal heath coordinators: two maternal health coordinators with an oversight role over child and maternal health services at the two study sites and the rest of Lilongwe District; one Coordinator responsible for the entire zone (comprising of 4 districts including Lilongwe); and one Coordinator at Ministry of Health, Reproductive Health Unit responsible for maternal services at National level. The key maternal care health workers were approached in their work stations through the nurses in charge of the respective health facility and the Ministry of Health Reproductive Health Coordinator who provided information about the study and referred them to the research team. Those willing were included in the study after the consenting process.

### Data collection

We conducted 22 in-depth interviews of perinatally depressed women (12 antenatal and 10 postnatal women) until we reached data saturation as well as 10 key informant interviews (6 Primary Health Care Workers and 4 Maternal Health Coordinators) from November 2017 to April 2018. Each interview lasted approximately one hour. Interviews were conducted by 3 Malawian interviewers: the Principal Investigator (PI), a female mental health nurse specialist, experienced in conducting qualitative research studies and two research assistants: JK, a male registered mental health nurse trained at graduate level and CT, a female community mental health nurse specialist. Although all the interviewers completed a Western mental health model of training, their long years of hands on experience in community mental health in Malawi, their cultural background and contextual knowledge and awareness enabled them to elicit in-depth information regarding perinatal depression and health care needs. Inclusion of a male interviewer in a maternal health related topic provided diversity and enriched our data. Each research assistant conducted the interviews separately with supervision from the PI after completing refresher training in qualitative research methods including conducting qualitative in-depth interviews. The investigators developed the open ended interview guides used in the study (Tables 1 and 2 provides a summary of interview guides. The full interview guides are included as supporting information). The interview guides were developed based on the study objectives and informed by the existing literature. The guides were pretested at Lumbadzi PHC clinic in October, 2017. Lumbadzi PHC clinic was not one of the study sites. The interview guides were subsequently modified in an iterative process until the study team was confident in the performance.

**Table 1:** A summary of interview guide for perinatal women.

We are concerned that you may be struggling with depression or feeling “not yourself” 1. What does depression mean to you?, How does it affect your functioning 2. What do you think has caused this problem? 3. Tell me about your childhood experiences 4. Tell me more about your experiences during this pregnancy/ delivery and postpartum? The challenges you met during pregnancy/ delivery and postpartum 5. Tell me more about your family experiences/family life? 6. Tell me about any experience of trauma, neglect or violence you have experienced; from spouse or from someone else in your life? How do these experiences affect you? 7. Can you tell me what you have been doing to cope with your challenges? 8. If you could have one thing to support you, what would it be? 9. What assistance do you think health care providers can offer you?

**Table 2:** A summary of interview guide for health workers and maternal coordinators.

1. What is your understanding of perinatal depression? 2. What is your view of perinatal depression? 3. Can you tell me impacts/complications of perinatal depression? 4. Are there any efforts made within safe motherhood initiative/Reproductive Health to address this problem? What is being done, by who? 5. What interventions do you think can best help women with perinatal depression? 6. Can such interventions be implemented in primary health care setting and/ the community? 7. Would such interventions be feasible and acceptable at Primary Health Care level or Community? 8. What would hinder provision of interventions for perinatal depression? Be it pharmacological or nonpharmacological?

All interviews with perinatal women were conducted in quiet and private rooms within maternity “waiting homes” at the two sites, counseling tents in Area 25, and offices for key informants. Key informant interviews were conducted in English while in-depth interviews with perinatal women were conducted in Chichewa (the local language) and translated into English. All interviews were audiotaped and transcribed. We recorded any significant observation and nonverbal ques during interviews and soon after data collection. Following the interviews, all 22 women were referred to psychiatric nurses for assessment and further management. All study participants were provided with refreshments in appreciation of their time and we provided an equivalent of 4.5 USD as transport and lunch reimbursement to perinatal women who participated in in-depth interviews.

### Data management and analysis

Data analysis was done concurrently with data collection and it was conducted according to the Consolidated Criteria for reporting Qualitative research (COREQ) [39]. To ensure credibility of this study, data was triangulated, we used multiple sources of data to elicit wider views of perinatal depression and health care needs i.e. perinatal women with depression, Primary Health Care worker and Maternal Health Coordinator. Multiple qualitative research experts were also used to analyze data to minimize potential bias in thematic coding [40].

All audiotaped interviews were transcribed verbatim and Chichewa transcripts were translated into English. The PI listened to all audio recordings and verified the 22 transcripts and the translation from Chichewa to English with the help of a language expert (JC). Qualitative content analysis was used to analyze the data [41]. The Principal Investigator read and reread notes and transcripts to become familiar with the data. To ensure reliability of coding and consistency, the PI and a third author, an expert in qualitative research (EC) independently read the first two transcripts line by line to identify and assign codes to similar concepts that repeatedly emerged from the data [42]. Through a consensus process we generated the first code list from the first two transcripts (there were similarities on all codes from the 2 coders). The first code list was reviewed and confirmed by an independent qualitative research expert (LMN) against a set of transcribed interviews, sampled audio recordings and interview guides. The first code list was modified following the review. The PI then coded the rest of the transcripts, with feedback from the senior authors, deleted repeated codes and added new ones until final code list was created. The final code list was agreed upon by joint consensus of all authors. Links between these codes were identified, repeatedly identified codes were merged, and themes and subthemes were generated from these codes. COREQ criteria has been used in presentation of the analyzed data [39].

### Ethical approval

Ethical Clearance for the study was obtained from the College of Medicine Research and Ethics Committee (COMREC) and permission to conduct the study in Area 25 PHC clinic and Kabudula rural hospital was obtained from Lilongwe District Health Office (DHO). Information sheets, containing study aims, expectations, benefits, possible risks and risk mitigation were read out to all potential participants confidentially, and an opportunity to ask questions was granted to ensure that they understood. Those who were able to read were given an opportunity to read the information sheet. Written consent (signature or thumb print verified by a witness) was obtained from the participants before commencement of the study. We also obtained consent to use audiotapes in interviews. Following the interviews, all 22 women were referred to psychiatric nurses for assessment and further management. We collected data from perinatal women with depression who were attending their routine antenatal and postnatal care. Despite their vulnerability, it is important that this population is investigated to understand their life experiences and views when considering an intervention.

## Results

### Demographic characteristics of perinatal women

Demographic characteristics of the perinatal women are summarized in Table 3.

**Table 3:** Sample description of perinatal women screened positive on EPDS (n = 22).

Demographics	Number
**Age in years**	
20 & below	8
21–30	10
31–40	3
41–50	1
**Marital status**	
Single	4
Married (monogamous relationship)	7
Married (Polygamous relationship)	8
Cohabiting	3
**No. of Pregnancies**	
First pregnancy	6
Two or more pregnancies	16
**Occupation**	
Employed	2
Not employed (Housewife and/or subsistence farmer)	20
**Source of income**	
Business	5
Farming & piece work	6
Salary	2
Husband	9
**Type of housing**	
Owned	9
Renting & care taker	13

### Characteristics of key informants

We recruited 10 key maternal care health workers. Six Primary Health Care workers and 4 maternal health coordinators. Six key maternal care health workers were females and 4 were males. Their age ranged from 24 to 50, they had their job positions for 2 to 9 years and their academic qualification ranged from a Certificate in clinical medicine to a general nursing or Master’s degree in reproductive health.

### Main findings

Data from Perinatal women and key informants from the two study sites was categorized in five major themes: (1) Awareness of depression, (2) Psychological and Social Challenges, (3) Support needs, (4) Health Service needs, and (5) Health care system strengthening. There were also fourteen subthemes that emerged from the data. This data will be presented together in a narrative format following the themes with supporting quotes.

#### Theme 1: Awareness of depression

We found that all perinatal women recognized and were able to describe the symptoms of depression they were experiencing. These were grouped under subthemes; disturbances in sleep and appetite and failure to function. Most women used a variety of terms and phrases to describe what they were feeling, i.e. “difficult times you are passing through”, “feelings of persistent worry”, “unsettled mind” and “heavy heartedness”.

**Disturbances in sleep and appetite**

Many perinatal women, (n = 17) reported feelings of sadness and having sleep problems due to persistent thoughts/thinking too much. Sixteen women reported loss of appetite, feeling full even without eating, difficulties in swallowing food and weight loss. Two of these women reported the experience of feeling as though something was blocking their swallowing:

*“Sometimes the food doesn’t go through*. . . .*yes*, *I force myself that I really have to eat today*, *I didn’t eat yesterday yet am not feeling hungry*. . . . . . . *It’s like there is a rock on my throat and my heart is hot*, *even when I want to eat I fail to swallow the food……*..*I also do not have appetite for food*, *it’s like I have a lot to contemplate in my mind even if there is food I don’t want to eat”* (Perinatal woman # 01, Area 25)

**Failing to function**

Many perinatal women, (n = 12) reported difficulties in performing activities of daily living, including caring of themselves and caring for their babies. Some reported preferring being on their own (social isolation). Ill health in pregnancy also affected some women’s ability to function effectively: frequent headaches, malaria, tightness of the chest and heart palpitations were a cause of worry for these women:

*“I don’t feel good*, *most of the time I don’t have strength to perform daily household chores…*.*mm*, *I see that my body is not feeling fine*. *I get worried now and again*. *Am unhappy…*. *most of the times I don’t eat when I start thinking a lot*, *or I don’t go to the farm*, *I just stay at home”* (Perinatal woman #21, Kabudula)

**Health worker understanding of depression**

Contrary to perinatal women, who explained depression through their experiences and how the experiences impacted their lives, key maternal care health workers (PHC Workers and maternal Health Coordinators) described depression as a mental health problem occurring during pregnancy and/or after childbirth. Some key maternal care health workers further described it as a mood disorder caused by social problems. It was considered a public health problem by all key maternal care health workers although they stated that it is rarely identified and managed:

*"I think the problem is common only that it is not recognized because of the short time women stay in the post-natal ward*, *I think that sometimes women start showing signs after discharge* [from maternity or postnatal ward]. *They might start maybe by refusing to breastfeed the time they are being discharged and we don’t know what happens thereafter*, *but I still feel its common only that we miss the opportunity to diagnose and treat those women”* (Maternal Health Coordinator # 7)

#### Theme 2: Psychological and social challenges

Most women (n = 16) reported that their psychological and social challenges were a primary cause of their problem/depression. This included general life struggles, relationship problems, fear of pregnancy outcomes and traumatic events.

**Life struggles**

Poverty, struggling to meet their daily needs, having no means of earning a living, having no food and poor living conditions were a source of worry for almost all women, (n = 20). While most perinatal women in rural Kabudula area had permanent homes within their villages, women in Area 25 were mostly staying in rented houses (n = 5) and four were staying in makeshift homes, a situation that caused a lot of stress to them. Most women, (n = 15) talked of having no peace of mind, having persistent thoughts about their problems and some (n = 10) expressed a wish to die or commit suicide:

“It’s just that when you think too much, you just feel that you are a failure, you think….. Maybe if I just left this world so that I should not meet these earthly problems again….Most of the time I think that I should just die when am giving birth and leave the child…I remember a certain time…. I would think about taking some medicine “overdose”,

(Perinatal woman # 05, Area 25)

**Relationship problems**

Inadequate and/or complete lack of support from the spouse especially material support such as clothes, food and money was a major cause of worry for majority of perinatal women, (n = 14). Some women (n = 6) reported feeling not cared for/not loved by their spouses:

*"What was really paining me was that when he took me*, *it was difficult to meet basic needs for the home*, *he could not help out at all*, *for me to find help I had to ask from my relatives"* (Perinatal woman #13, Kabudula)

Women in polygamous relationships (n = 6) reported that lack of support was related to their spouse’s decision to take another wife/wives. Polygamy was common in the rural setting (Kabudula) where six women were in polygamous relationship compared to two in Area 25, an urban setting. These women expressed feelings of betrayal and stressed as they now have to compete for attention and resources.

*"… These others* [pregnancies] *were just ok*. *When I got pregnant I could tell him that am pregnant*, *the love was there and he would do everything necessary for me but now things have changed because of this…… the new wife……the care is reduced compared to the way things were in the past*, *that’s why you end up being depressed”* (Perinatal woman #01, Area 25)

Infidelity, abandonment and conflicts were also identified as some of the causes of depression. Eight women were affected by their spouse’s unfaithfulness, infidelity was also reported as a source of frequent conflicts within the women’s relationships. Young women aged 18 to 20 years (n = 5) who dropped out of school after becoming pregnant, were struggling with issues of abandonment and rejection by their partners and parents/guardians. They expressed having no peace in their minds and feelings of guilt, self-blame, regret and hopelessness;

*“The problem started when I realized he has a girlfriend*. *He sleeps around with other women………his behavior worries me*, *I am concerned and worried about my future*. *I worry about what’s going to happen in the future*. . . . . . *I regret getting pregnant*, *it pains me because I was not prepared for it [the pregnancy]*, *I wanted a bright future and I wanted to get educated…*..*”* (Perinatal woman #13 Kabudula)

**Fear of pregnancy outcome**

The majority of the pregnant women (n = 13) expressed worry and had fears regarding birth outcomes. Witchcraft and ancestral beliefs of having mixed blood if one has had more than one sexual relationship was also a source of fear among a few women who thought they would have problems during delivery due to such beliefs.

Uncertainty about labor and delivery processes affected prim gravidas. They stated that no information on labor and delivery was provided to them at the antenatal clinics. Whereas some participants (n = 7) were afraid because of previous negative experiences:

(Previous pregnancy*)………“They* [Doctors] *said the way the situation is*, *either me or the unborn baby will die*, *so after the operation the baby survived and luckily I survived……*.*they used to get me off the bed*, *I had a catheter*, *I couldn't even walk*,*……… Now I am affected*, *I am always in fear thinking that what happened last time is going to happen again*. *I am never at peace*, *sometimes am just very quiet*, *because am praying all the time”*

(Perinatal woman #09, Kabudula)

**Traumatic events**

Some participants expressed feelings of emotional pain described as heavy heartedness, heartache, frustration, or sadness due to traumatic events that they had previously experienced or were experiencing such as loss of previous pregnancy or baby and various forms of abuse from their partners. Eight women reported experiencing intimate partner violence:

*"……days went by and we were still together with the other woman like two wives in the same house… I was removed from the bed and started sleeping on the floor on top of clothes*, *sometimes he would hit me*, *sometimes she* [the first wife] *would tell me that if I think the pregnancy is a burden I should just terminate it*, *therefore I realized that I could not cope and I left……*.*"* (Perinatal woman #05, Area 25)

Similar to the perceptions of the perinatal women, the key maternal care health workers also identified stressful life events including marital issues (polygamy, infidelity, lack of support, abandonment/rejection and divorce) as well as poverty, loss of baby or loved ones as causes of depression. Key maternal care health workers also reported a positive history of mental illness, other health issues, HIV, unwanted pregnancy and gender of a child as causes of depression. Some key maternal care health workers reported having been approached by women who wished to terminate their pregnancies due to various reasons including pressure from their spouses:

*"Sometimes an unwanted pregnancy may lead to that* [depression], *sometimes there are family issues*, *sometimes a woman might have a history of mental illness and relapses*. *Also poverty*, *as you know area 25 PHC clinic is surrounded by a communities with low social economic status*, *especially area 50*, *you talk of Ngomani area*. *In those areas most women are not empowered*, *they have low education level*, *they depend on their husbands*, *so the husbands can do anything because they know that this one cannot leave so it’s really a challenge"*. (Primary Health Care Worker # 1)

#### Theme 3: Support needs

Coping strategies used by perinatal women ranged from spirituality, seeking help from others and keeping busy or working hard.

**Spirituality**

Prayer and participating in religious activities was reported as a major way of coping by some (n = 13) perinatal women. Women reported having hope and experiencing relief from their issues through following prayer activities and religious counseling:

*"I like to associate with religious groupings, to lessen my problems. When people are praying, I hear some messages that gives me courage, I told church counselors about my problem, right now they come to visit me to find out how I am coping"*.

(Perinatal woman #12, Kabudula)

**Support from others**

Some perinatal women (n = 11) expressed need for financial and material support from their partners, family members or any other well-wishers. They were of the view that all their problems would be gone if they had money or means of earning money e.g. a steady business. A few perinatal women (n = 9) expressed a willingness to rebuild their relationships with spouses and in-laws and a need for encouragement/advice on how to live their lives and deal with problems. Two women expressed need for partner’s support or help with care of a baby:

*"The areas I would want to change most is ……if I can find some money to start a business*. *I think that I would manage to solve some problems in my life because I cannot find money from my husband*, *I have been trying*. . . .*"* (Perinatal woman #03, Kabudula)

#### Theme 4: Health care needs

The majority of perinatal women were not sure of the services they would access from the health care workers/the health facility.

**Psychological interventions**

When asked about management options available for depression, most perinatal women (n = 19) indicated that they would take part in counseling, thirteen stated that they would prefer individual counseling and a few (n = 6) were in support of group counseling. Some women expressed uncertainty regarding effectiveness of group counseling, they stated that it would be difficult to communicate and understand each other in a group setting; others felt it would be culturally inappropriate to share personal and family problems with other people in a group as this might cause problems with their in-laws while some were concerned with confidentiality of their personal information by fellow group members:

*“I cannot accept talking about my own problems in a group*. *I would talk about them [problems] on one to one level*, *then I would go in a group to learn more………*.. *I have seen before*, *some other people*, *you tell them something*, *they share the issue somewhere else with others and add some lies too "*. (Perinatal woman # 21 Kabudula)

Psychoeducation about depression and problem solving skills was also seen as a way to help them prevent depression or cope with their problems. Some perinatal women expressed the wish for health workers to offer support and take time to talk to them in antenatal as well as postnatal clinics. Most perinatal women were not in favor of taking medication while they were pregnant or breast feeding in fear of affecting the baby.

On the other hand, creating awareness about perinatal depression in the community and amongst health care workers and receiving appropriate training were identified by key maternal care health workers as central to providing care to those affected:

*"Before we do community sensitization it would be good to sensitize the health care providers so that they are able to identify if a woman has depression and then sensitize the community so that they themselves can identify if they have depression*. *Family members around can also identify and help in seeking care*. *Health care workers should also be well knowledgeable to be able to help"* (Maternal Health Coordinator #5)

**Family/partner involvement**

Some perinatal women stated that involving their partners will help them work through their problems and rebuild their relationship. Similarly, key maternal care health workers indicated that family and or partner involvement would be significant in managing perinatal depression:

*"We miss the opportunity of male involvement because we introduced the concept of male involvement* [in maternal health care] but *the way we are involving our men…*.*we miss a lot of opportunity because there is no standard*, *so unless there are specified antenatal interventions that could be incorporated in antenatal care so that when a man escorts a woman*, *at least you should not miss the psychological aspect*, *we should take advantage of the male involvement because the man is key”*. (Maternal Health Coordinator #7)

#### Theme 5: Health systems strengthening

Key maternal care health workers expressed a need for a review of some systems and processes i.e. human resource, they recommended utilization of other staff cadres in managing women with perinatal depression (task shifting) and strengthening health care delivery systems in order to effectively manage women with perinatal depression i.e. integrating depression screening in existing screening tools, developing treatment guidelines and establishing a clear service pathway;

**Personnel**

Most key maternal care health workers attributed the lack of focus on maternal mental health issues to their heavy work load. They indicated that it is possible to manage maternal depression provided there is involvement of other cadres whether skilled or nonskilled workers (task shifting) and that proper training, supervision and incentive should be provided for these workers.

*“Everything is possible but if you engage the nurses they are already overwhelmed but maybe use the volunteers as HIV Testing Counselors are used*, *just train them and of course train the nurses and the clinicians so that they should supervise*. *…*, *as long as they are given a little something* [some incentives]*”* (Maternal Health Coordinator #7)

While some perinatal women stated that they failed to approach health workers because clinics are always full, others (n = 7) expressed fear in approaching health workers with their problems in the clinics due to the negative attitude usually displayed by the latter. These participants expressed a need to engage mature and experienced providers who are ready to offer them support. They also suggested utilizing existing community structures and volunteers like “community secret mothers” (community volunteers who work with pregnant women in confidence with an aim of initiating early antenatal care and encouraging hospital delivery) who can facilitate groups in their communities:

*“At first there is need for someone from the hospital to teach the volunteers and from these volunteers one can continue leading the group to give time to [relieve] health workers ……*. *And visiting them at intervals……*. *You* [hospital staff] *should also be having a day to visit the groups…………"* (Perinatal woman #08, Area 25)

**Health care delivery system**

The lack of maternal mental health guidelines was also identified by key maternal care health workers as a major hindrance in providing maternal mental health services including depression management. Most key maternal care health workers expressed need for incorporating depression screening in the existing screening tools and development of service pathway and guidelines:

*"I think the key is integration because it* [management of perinatal depression] *will definitely be seen as an extra burden*, *so many initiatives introduced years ago are considered burdensome and they haven’t really been integrated*. *We already have so much work*, *so much paper work it’s really hard*. *So if it was to be integrated it would have to be in the same admission form that we have*. *Let’s say incorporating a checklist of signs of depression in antenatal period and some questions can be asked before discharge* (in postpartum), *and when they* [the women] *come for postnatal check-up to identify cases of depression"*

(Maternal Health Coordinator #5)

Key maternal care health workers were also of the view that an Intervention to manage perinatal depression would be easily accepted if there is collaboration among all stake holders. Whether its community or hospital based intervention it needs to be nested within the existing health care delivery system.

## Discussion

This is the first qualitative study conducted in Malawi to assess the perceptions of perinatal women with depression and health care workers providing maternity care. This present study extends in important ways the only prior qualitative study in Malawi that examined perceptions of childbearing women about perinatal distress and depression [29] and it increases our understanding about perinatal depression. The present study sought perceptions of perinatal women with depression and key maternal care health workers (PHC workers and Maternal Coordinators), and found the various challenges perinatal women with depression encounter, how they cope with these challenges and the need for treatment and support. The present study also identified gaps in the health care system that were hindering provision of care i.e. shortage of staff, lack of awareness and capacity to identify and manage depression, lack of treatment guidelines and service pathways. The involvement of key maternal care health workers in the present study is vital as their perceptions will inform dialogue about treatment practices and influence policy change.

Perinatal depression was identified as a major health concern by all study participants and they acknowledged the need for an intervention that will address the identified challenges. Additionally, they viewed strengthening the health delivery system as crucial to effectively address the health care needs of perinatal women and gaps identified in the system. Awareness creation, task shifting, capacity building, integration of depression management in existing screening tools and inclusion of maternal mental health guidelines in reproductive health care were recommended as tools to effectively address perinatal depression.

### Experiences of perinatal women with depression

Previous studies in Sub-Saharan Africa have reported symptoms of depression similar to those described in international criteria, i.e. ICD 10 and DSM V (27, 28, 30). Similarly, we found that most symptoms reported by perinatal women were similar to these studies and international classificatory systems. Perinatal women used culturally applicable phrases/expressions/idioms to describe their experiences of distress. Idioms of distress have been described as social and cultural ways of expressing distress [43]. These findings are consistent with those reported in other studies conducted in South Africa and Nigeria [27,30]. This highlights the significance of identifying different terminology and idioms used for depression in local communities in Malawi and incorporating these in research and depression management, in line with recommendations from a previous systematic review [44].

Depressive symptoms affected self-care activities and the women’s functioning. These findings are consistent with what was previously reported in a South African study [45]. Traditionally in an African setting, women perform caretaking roles and failure to perform such roles might be a source of shame and additional stress. The reported emotional disturbance and inability of the woman to take care of herself and the baby will consequently affect child growth and development, nutrition and attachment [21,22,24]. Hence, this highlights the significance and need for early identification and management of maternal depression to promote the health of the mother and the baby, as recommended in studies elsewhere [46].

Maternal depression has also been associated with history of stressful life events in previous research [9]; similarly, this was the case in this present study. The majority of the perinatal women interviewed were not employed and were not engaged in any financially gainful activity at the time of the interview. These women depended on their partners and other family members for financial support. A previous Malawian study found that support from the partner was viewed as crucial in successful delivery and raising a healthy child [29]. In a traditional Malawian family, men are regarded as providers of financial support and protection. Consequently, the lack of support, both emotional and material reported in this study primarily affected the women. These findings are consistent to those reported in previous studies [27,28,31,47]. Women in polygamous relationships faced an added challenge of competing for attention, love and resources. This was not only a source of conflict in the relationship, it also brought about shame and disappointment in women. Polygamy was very common in the two study sites. Previous studies conducted in Uganda and South Africa [28,31] also identified similar challenges faced by women in polygamous relationships.

Intimate partner violence has been reported to have a significant adverse impact on mental health [48,49] and consistent with other studies, intimate partner violence and other forms of abuse are serious relationship problems that were perceived as causes of depression in this study. Because of lack of effective support systems, it might be that these women were struggling to deal with these challenges on their own. Even those with support might have had difficulties seeking help from others. This is likely because marital issues are sensitive issues in nature and are supposed to be handled by marriage counselors within the family unit in the Chewa culture.

Similarly, previous negative experiences and fear of birth outcomes triggered strong feelings of emotional pain for most pregnant women especially when they found themselves at a period of uncertainty as reported in a Malawi study [29]. This issue calls for emotional support and education for first time mothers and women with known previous birth complications.

### Service needs

Our findings showed that information and education on the process of labor and delivery was an important need for women with a first pregnancy. Information about depression in the perinatal period and counseling was also identified as a need to help women prevent and cope with their experiences. This is consistent to similar unmet needs identified in a Ugandan study [31]. In our work, although these women were attending antenatal care, there was inadequate information and attention given concerning their specific situations. This was compounded by a lack of availability of health workers due to their workload and negative attitudes. While shortage of health workers and negative attitudes has been found to be the greatest barrier to accessing services in health facilities in LMIC [31,50], lack of knowledge on the condition was also identified by key informants as contributing greatly to their inability to provide necessary help to women in this study. This issue is likely due to the fact that the mental health training course is a smaller component in pre service training courses for most health related programmes in Malawi, and with lack of practice and exposure most health workers completely lose the competency for diagnosing and treating mental illness. Our results replicate those found in a Ugandan Study [31] and therefore highlight the need to incorporate maternal mental health training in primary health care that can stimulate wide scale training programmes.

Low staffing level and inadequate training is a big problem in health care systems in LMIC [50–52]. This affects universal health coverage and the quality of care provided. Key maternal care health workers in this study indicated that the current PHC staff (nurses and clinicians) would not be able to provide interventions for perinatal depression due to work overload. We found that one midwife was responsible for more than 30 women in antenatal and postnatal clinics in a day and would attend to more than 8 deliveries per night. Staff in maternal and child health services spend very little time with perinatal women hence missing the opportunity to identify and manage those with maternal mental illness. Similar results were reported in studies conducted in Uganda and Ethiopia [31,53]. Consequently, there is a need to seriously consider task sharing to bridge this treatment gap. Task sharing/shifting where trained non-specialist and lay health workers can deliver an intervention which would otherwise be delivered by specialist workers [54] has been recommended to be an effective and more feasible approach to increase human resource for mental health and expand mental health coverage [55]. Studies elsewhere have reported effective interventions implemented by nonskilled workers and community volunteers in LMIC [26,56–58].

### Intervention

The psychological and social challenges found in this study necessitates implementation of a family focused psychosocial intervention that would address psychosocial challenges and information needs of perinatal women through pregnancy, delivery and caring for a baby while empowering the women in problem solving.

Contrary to findings reported in previous studies regarding the effectiveness and acceptability of group counselling/interventions for perinatal women with depression in LMIC (for instance, peer-led parenting intervention in Lira, Uganda [59] and multicomponent intervention for postnatal women in Chile [60]), most perinatal women in this study preferred individual counseling. They were concerned with confidentiality issues, cultural appropriateness of discussing personal and family issues in a group setting and the impact this might have on their family especially in-laws. This might be due to the women’s concern/fear of being stigmatized in the community or lack of information regarding operation of group counseling. Our findings also show that the majority of perinatal women were not sure of the services they would access from the health care workers/the health facility. This calls for more research in this area and awareness creation on perinatal depression as a health problem and its treatment options. These findings also suggest consideration of an intervention that can be provided to individual women and their families in this setting.

Interventions like the “Thinking Healthy Programme (THP)”,administered to individual women and family members in their homes with support group sessions, can address several issues identified in this study i.e. women concerns of confidentiality, cultural appropriateness of discussing family issues in a group session, and fears women have to approach health workers in hospital setting. THP, is a Cognitive Behavior Therapy (CBT) based intervention developed and tested in a low resource setting (Pakistan), it address the physical and psychological health of perinatal women and the mother-infant relationship through general principles of care, psychoeducation and counseling [26]. THP was adopted by WHO as a first line low intensity psychosocial intervention for perinatal depression [61] and it does not require previous knowledge or experience of mental health care [25]. Such interventions have not only proven effective in reducing depression symptoms, they have also shown that it is feasible and acceptable for nonskilled workers to implement such services in LMIC [26,62]. THP has been adapted for use by lay health workers and peer volunteers (Thinking Health Programme-Peer Delivered, THPP) [26]. In the present study, we found that perinatal women and health workers recommended the use of community volunteers including the use of existing structures like “secret mothers”. Secret mothers are mature women volunteers who work with pregnant women in some communities with an aim of initiation of early antenatal care and encouraging hospital delivery. Such women volunteers can be a great resource in the face of shortage of health workers identified in this study and perinatal women’s preference of “mature and experiences women”.

Psychological interventions are recommended and an appropriate first line treatment for perinatal depression. Women with moderate to severe depression may be reluctant to accept medication when pregnant and breastfeeding [54,63]. Similarly, in this study, we found that most women were not in favor of taking medicine and they did not consider their problems an illness that warranted medication. Rather they regarded their symptoms as social problems related to difficulties they were experiencing, thus, confirming previous findings that have been reported in the literature [64,65]. Spirituality, prayer and religious counseling was used by most women as a coping mechanism in this study. These findings suggest that more research is required to evaluate the effectiveness of such strategies for women with depression and assess feasibility and acceptability of psychological interventions for women in this setting.

Health workers in our study were of the view that screening for maternal mental health problems is feasible provided it is integrated in existing screening tools and that interventions can easily be accepted in the community through integration and collaboration from Ministry of Health down to health facilities. Integrating depression screening and management in existing screening tools has been shown to narrow the treatment gap [56].

### Limitations

This study had several limitation that needs to be considered when interpreting the findings, for instance we did not interview some perinatal women (n = 5) who were referred for diagnostic assessment and management urgently because of the severe nature of their condition. Perceptions of these women were not included, hence the findings might not be representative of all perinatal women with depression.

We interviewed 10 key informants until we reached data saturation. As in all qualitative studies, we intended to gain deep insights of perinatal depression and health care needs rather than generalizing the findings.

This study was conducted in only one region of Malawi and one of the sites (Kabudula Rural Hospital) was dominated by a culture (Chewa) where expression of personal and family issues by perinatal women was limited. However inclusion of an urban setting (Area 25 PHC Clinic) enabled us to have contact with perinatal women from different regions of Malawi and with diverse culture and views.

This study focused on a sample derived from public health facilities, which might not be representative of the rest of perinatal women accessing care in charity-run or private health facilities. Nonetheless, our study findings are consistent with other African studies and build on findings of a perinatal distress and depression study conducted in Malawi.

## Conclusion

We found that perinatal depression is recognized as a common mental health problem although it is often missed and not managed appropriately in PHC clinics in Malawi. Our study also revealed the complex psychosocial issues affecting perinatal women with depression and the challenges in the health care system that hinder maternal mental health services delivery. This work calls for a need to strengthen the health delivery systems especially at the primary care level where the majority of women access care. Further, it provides support for plans to develop/adapt an intervention that focuses on the expressed needs of women (psychological, social as well as informational needs), involves family members, and can be implemented by nonskilled workers in the community.

## Supporting information

S1 File Qualitative data collection tool. (ZIP)
