# Supplementary material for: “Passing through difficult times”: Perceptions of perinatal depression and treatment needs in Malawi - A qualitative study to inform the development of a culturally sensitive intervention
Source: PLoS One. 2019 Jun 18;14(6):e0217102. doi: 10.1371/journal.pone.0217102 (PMC6581242; doi:10.1371/journal.pone.0217102)

**CODE NO: ..................................**

**Edinburgh Postnatal Depression Scale (EPDS) – Modified English version**

I am going to ask you some questions about how you have been thinking and feeling during the past one week. For the first 2 questions we will use side 1 of the card. Each picture represents one of the 4 possible answers. I will point to them as I read the choice of answers for each question. You should choose which answer applies to you over the last 1 week.

| 1 | In the last week, have you been able to laugh  and see the funny side of things? | [3] Not at all  [2] Definitely not so much now  [1] Not quite so much now  [0] As much as you always could |
| --- | --- | --- |
| 2 | In the last week, have you looked forward with enjoyment to things? | [3] Not at all  [2] Definitely not so much now  [1] Not quite so much now  [0] As much as you always could |

For the rest of the questions we will use side 2 of the card. Again, each picture represents one of the 4 possible answers. I will point to them as I read the choice of answers for each question. You should

| 3 | In the last week, have you blamed yourself unnecessarily when things went wrong? | [3] Most of the time  [2] Some of the time  [1] Not very often  [0] Never |
| --- | --- | --- |
| 4 | In the last week, have you been anxious or worried for no good reason? | [3] Very often  [2] Sometimes  [1] Hardly ever  [0] Not at all |
| 5 | In the last week, have you felt scared or panicky for no very good reason? | [3] Yes, quite a lot  [2] Yes, sometimes  [1] No, not much  [0] No, not at all |
| 6 | In the last week, have things been getting on  top of you? | [3] Most of the time you haven’t been able to cope at all  [2] Sometimes you haven’t been coping as well as usual  [1] Most of the time you have coped quite well  [0] You have been coping as well as ever |
| 7 | In the last week, have you been so unhappy that you have had difficulty sleeping? | [3] Most of the time  [2] Quite often  [1] Very often  [0] Not at all |
| 8 | In the last week, have you felt sad or miserable? | [3] Most of the time  [2] Quite often  [1] Not very often  [0] Not at all |
| 9 | In the last week, have you been so unhappy that you have been crying? | [3] Most of the time  [2] Quite often  [1] Only occasionally  [0] Never |
| 10 | In the last week, has the thought of harming yourself occurred to you? | [3] Most of the time  [2] Quite often  [1] Only occasionally  [0] Never |

**CODE NO: ..............................**

**Edinburgh Postnatal Depression Scale (EPDS) - Chichewa version**

| 1 | *Masiku asanu ndi awiri apitawa,* kodi mwakhala mukutha kuseka komanso kuona kusangalatsa kwa zinthu? | ❒ [3] Olo mpang’ono komwe  ❒ [2] Panopa osati kwambiri  ❒ [1] Osati bwino kwambiri  ❒ [0] Monga m’mene mumathera nthawi zonse |
| --- | --- | --- |
| 2 | *Masiku asanu ndi awiri apitawa,* kodi mwakhala mukudikira ndi nsangala mu zinthu zozachitika mtsogolo? | ❒ [3] Olo mpang’ono komwe  ❒ [2] Panopa osati kwambiri  ❒ [1] Osati bwino kwambiri  ❒ [0] Monga m’mene mumathera nthawi zonse |

Tsopano ndikufunsani mafunso am’mene mwakhala mukuganizila ndikumvera masiku asanu ndi awiri apitawa. Mafunso awiri oyambilira tigwilitsa ntchito mbali imodzi ya kadi. Chithunzi chilichonse chikuimila limodzi mwa mayankho anayi. Ndidziloza zithunzi ndikamawelenga mayankho a funso lililonse. Musankhe yankho logwilidzana ndi m’mene mwakhala mukumvela masiku asanu ndi awiri apitawa.

Mafunso otsatilawa tigwilisa ntchito mbali yachiwiri ya kadi. Mobwelenzanso,chithunzi chilichonse chikuimila limodzi mwa mayankho anayi. Ndiziloza zithunzi ndikamawelenga mayankho a funso lililonse. Musankhe yankho logwilizana ndi m’mene mwakhala mukumvela masiku asanu ndi awiri apitawa

| 3 | *Masiku asanu ndi awiri apitawa,* kodi mumazida nokha mosafunikila pamene zinthu sizinayendebwino? | ❒ [3] Nthawi zambiri  ❒ [2] Kawirikawiri  ❒ [1] Mwakamodzikamodzi  ❒ [0] Sizinachitikepo |
| --- | --- | --- |
| 4 | *Masiku asanu ndi awiri apitawa,* kodi mumakhumudwa kapena kudela nkhawa popanda chifukwa chenicheni? | ❒ [3] Kwambiri  ❒ [2] Nthawi zina  ❒ [1] Sizimachitika  ❒ [0] Olo mpang’ono pomwe |
| 5 | *Masiku asanu ndi awiri apitawa,* kodi mumachita mantha kapena kusowa mtendere popanda chifukwa chenicheni? | ❒ [3] Kwambiri  ❒ [2] Nthawi zina  ❒ [1] Osati kwambiri  ❒ [0] Ngakhale pang’ono |
| 6 | *Masiku asanu ndi awiri apitawa,* kodi mwakhala mukuganiza kapena kumva ngati munalindi zinthu zambiri zoyenela kuchita koma simumakwanisa kuchita ? | ❒ [3] Nthawi zambiri mwakhala mukulepheratu  ❒ [2] Nthawi zina mwakhala mukulepheratu  ❒ [1] Nthawi zambiri mwakhala mukutha  ❒ [0] Mwakhala mukutha ngati m’mene mumapangira nthawi |
| 7 | *Masiku asanu ndi awiri apitawa,* kodi mwakhala osasangalala moti mwakhala mukulephera kugona? | ❒ [3] Nthawi zambiri  ❒ [2] Kawirikawiri  ❒ [1] Osati kawirikawiri  ❒ [0] Mpang’ono pomwe |
| 8 | *Masiku asanu ndi awiri apitawa,* kodi munali wokhumudwa kapena kusowa mtendere wa mumtima? | ❒ [3] Nthawi zambiri  ❒ [2] Kawirikawiri  ❒ [1] Osati kawirikawiri  ❒ [0] Mpang’ono pomwe |
| 9 | *Masiku asanu ndi awiri apitawa,* kodi mwakhala osasangalala moti mwakhala mukulira? | ❒ [3] Nthawi zambiri  ❒ [2] Kawirikawiri  ❒ [1] Mwakamodzikamodzi  ❒ [0] Sizinachitikepo |

**EDINBURGH POSTNATAL DEPRESSION SCALE PICTURE CARD**

**SIDE A**


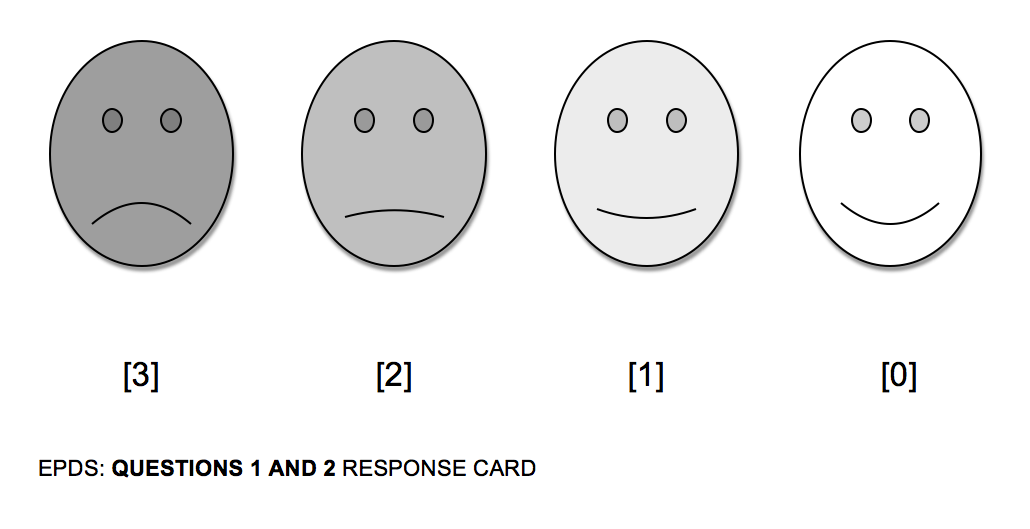


**SIDE B**


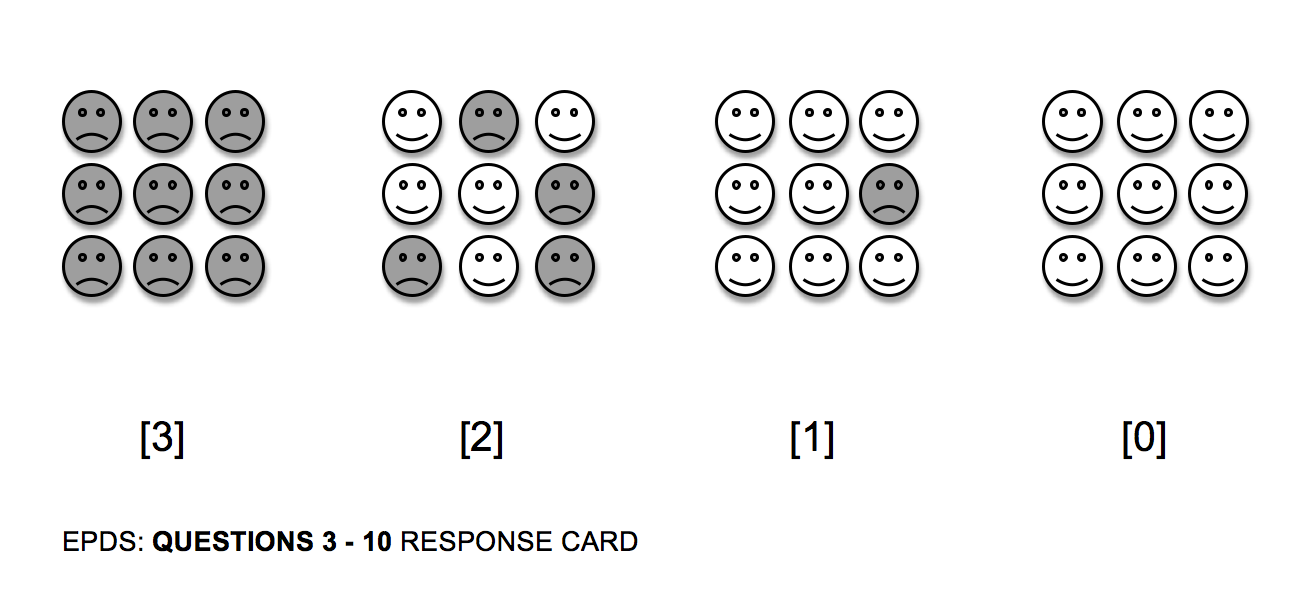

Supplement: S1 File — (ZIP) [file pone.0217102.s001.zip › Qualitative Data Collection tool/EPDS_ENGLISH AND CHICHEWA VERSION.docx]
