# Supplementary material for: “Passing through difficult times”: Perceptions of perinatal depression and treatment needs in Malawi - A qualitative study to inform the development of a culturally sensitive intervention
Source: PLoS One. 2019 Jun 18;14(6):e0217102. doi: 10.1371/journal.pone.0217102 (PMC6581242; doi:10.1371/journal.pone.0217102)
Supplement: S1 File — (ZIP) [file pone.0217102.s001.zip › Qualitative Data Collection tool/INTERVIEW GUIDE - PERINATAL WOMEN CHICHEWA.docx]

**PERINATAL DEPRESSION STUDY INTERVIEW GUIDE – PERINATAL WOMEN, CHICHEWA**

**Ndondomeko ya kafukufuku**

**Mafunso ogwilitsa ntchito kwa a zimayi apakati ndi omwe ali ndi khaNda la masabata atatu mu kafukufukuyu**

**Kwa ofufuza: akumbutseni otenga nawo mbali za kafukufukuyu, funsani ngati apeleka chilolezo choti mutha kuwafunsa mafunso/kukambilana nawo ndinso kujambula mayankho awo**

**Gawo loyamba: Mbili yanu**

1. Muli ndi zaka zingati?
2. Muli pabanja? Ku banjako mulipo akazi angati? Inu ndinu achingati? Banja ili ndi lachingati? Ngati munakwatiwapo kale kwina, chinachitika ndi chani kuti banjalo lithe?

Ndinu osakwatiwa? Ndinu oferedwa? Kapena banja lanu linatha?

1. Munayimapo kangati? Munabeleka kangati? Mimbayi/mimba ya mwana uyu yinali yokonzekela? Ngati ayi mumamva bwanji mumtima za mimbayi
2. Ana amoyo ndiangati? Akufa ndiangati? Mukudziwa chomwe chinadzetsa infa ya mwana wanu/za ana anu? Imfa zimenezi zinakhudza bwanji moyo wanu
3. Ndinu achipembedzo chanji?
4. Mumakhala kuti? Mumakhala nyumba yotani? Munyumbamo mumakhalamo anthu angati?
5. Mumagwira ntchito yanji? Ngati simugwira ntchito tsiku lanu mumaligwilitsa ntchito bwanji/mumatani tsiku lonse?

Chithandizo cha pa khomo mumachipeza bwanji?

**Gawo lachiwili: Mafunso okhuza vuto la kukhumudwa mu uchembele**

Ife ndife okhudzidwa kuti mwina mukuvutika ndi vuto la kukhumudwa/simuli momwe mumakhalila mmbuyomu

1. Vuto lokhumudwa limatanthauza chani kwa inu/ mumalimvetsa bwanji?

**Fufuzani:** mungafotokozepo momwe mwakhala mukumvera mumtima mwanu? monga kusangalala kapena kudandaula,

Mumangona bwanji usiku? Kodi mumavutika kuti mupeze tulo? Kodi mumadzukadzuka usiku? Kodi mumavutika kukhala ntulo usiku? Kodi tulo limakutherani mmamawa kwambiri Kapena mumagona kopitilira muyeso?

Chilakolako chanu pa chakudya chinali bwanji mu masiku asanu ndi awiri apitawa? Mukayerekeza ndi nthawi zonse, chilakolako chanu chinali bwanji? Kodi mumachita kudzikakamiza kuti mudye? Ndifotokozeleni zokhudzana ndi madyedwe anu - mumadya kwambiri kapena pang’ono, kuyerekeza ndi momwe mumadyera nthawi zonse, Nanga zimenezi zimachitika pafupifupi tsiku lirilonse?

Tandiuzeni mmene mukuwonera mphamvu zanu? Kodi mumakhala otopa nthawi zonse? Kodi mumakhala otopa pafupifupi tsiku lililonse,

Tandifotokozeleni zakusintha kulikose kokhudzana ndi masangalalidwe anu? Munakhalapo opanda chidwi kapena kukhala osasangalatsidwa ndi zinthu zomwe mumasangalatsidwa nazo nthawi zonse? **Ngati ndichoncho**: Kodi zimachitika pafupifupi tsiku lililonse? Zinachitika kwa nthawi yaitali bwanji? Kodi zinachitika mpaka masabata awiri?

Tandifotokozeleni kuti maganizo anu amakhala otani zinthu zikafika poyipa? **Fufuzan**i mumaganizira kwambiri za imfa kapena kuti bola mutangofa? Kodi munaganizako zozipweteke kapena kudzivulaza nokha?

**Ngati ndichoncho**: Kodi munayeserako kuzipweteka kapena kudzivulaza nokha?

Kodi mumadziwa kuti muli ndi vutoli?

**Fufuzani:** Kodi munadziwa bwanji? Alipo wina mmbanja mwanu/mmudzi mwanu kapena wa chipatala amene anakhuzidwa ndi vuto lanu nakuuzani?

Kodi kukhumudwa/kudandaula kwambiri kwabweletsa kusintha kwanji pa moyo wanu. **Fufuzaninso:** kuvutika kugwira ntchito yanu, kusamala mwana, kusamala zinthu za mnyumba mwanu, kapena kugwirizana bwino ndi anthu ena?

1. Mwakuganiza kwanu, mukuwona ngati chinayambitsa vutoli ndi chani?
2. Mundifotokozelepo za umwana wanu;

**Fufuzani:** mmene anakulira, mavuto omwe amakumana nawo akukula, ubale/ubwenzi wawo unali bwanji ndi abale, matenda alionse amene amavutika nawo, kuphatikiza matenda a misala,

Ngati panali zovuta zina **fufuzani** kuti zovutazi zakhuza bwanji moyo wawo

1. Ndifotokozeleni za momwe mwakhalira nthawi yino yauchembele

**Fufuzani:** mavuto amene amakumana/akukumana nawo nthawi yoyembekezela, momwe amachila, kapena pomwe ali ndi khanda.

Mavuto a mthupi, a mmaganizo, kuzisamala kapena kusamala mwana, kusowa zipangizo ndi ena

**Fufuzaninso:** mmene akukhalira mbanja

Ubale wawo ndi amuna awo/bambo a mwana, ndinso abale ndi abwenzi ena

thandizo lomwe amalandila kuchokela kwa amuna awo/abambo a mwana; monga zipangizo, kuwathandiza pochita zinthu/kugwira ntchito kapena kuwathandiza akakhala ndi malingaliro/maganizo.

Thandizo lochokela kwa amuna anu/abambo a mwana limakukwanilani/ndilokwana

1. Kodi munachitilidwapo nkhanza ya mtundu wina uliwonse (kunyozedwa, kusakusamalani, kugwiliridwa, kapena kukuvulazani) ndi amuna anu/abambo amwana kapena ena achibale.

**Fufuzani:** Kodi izi zimakhudza bwanji moyo wanu watsiku ndi tsiku

1. Ndifotokozeleni njila zomwe mwakhala mukugwilitsa ntchito kuti mupeputse mavuto mwandifotokozelawa.

**Fufuzani:** kufuna chithandizo kuchokela kwa amuna anu/abambo a mwana, abale, abwenzi, aphungu a ku chipembedzo ndi aphungu a mmudzi kapena a nkhoswe

**Fufuzaninso** thandizo lomwe amapeza kuchokera kwa anth awatchulawa

Nanga ndi njila zanji zina zomwe mumagwilitsa ntchito pozithandiza nokha

1. Kodi munakakonda mutalandila thandizo lanji kuchokela kwa ena?

**Fufuzani:** kuchokela kwa a muna anu/abambo amwana, banja lanu, kwa abale, abwenzi, ku ntchito, kuchokela kwa anthu a mmudzi, Kapena kuchipatala

1. Mukuganiza kwanu, mukuona ngati achipatala angapeleke thandizo lotani.

**Fufuzani:** mungalole kulandila/kutenga nawo mbali mu uphungu? **Fufuzaninso**: ngati angasangalatsidwe ndi uphungu wopelekedwa paokha kapena pagulu

**Fufuzaninso ngati angasangalatsidwe kulandila uphungu ku mudzi?** Mwakuwona kwanu munthu woyenela kupeleka thandizo limeneli kumudzi angakhale wotani? Mungakonde thandizo lopelekedwa pagulu kapena panokha

Mungalole kulandila mankhwala othandiza vuto la kukhumudwa?

Pangakhale chodetsa nkhawa chilichonse potenga nawo mbali muuphungu kapena kulandila mankhwala?

Kodi banja lanu lingachilandile bwanji ngati inu mwaganiza kufuna thandizo pa vuto lanu lokhumudwa kapena kutenga nawo mbali mu uphungu?
