# Supplementary material for: “Passing through difficult times”: Perceptions of perinatal depression and treatment needs in Malawi - A qualitative study to inform the development of a culturally sensitive intervention
Source: PLoS One. 2019 Jun 18;14(6):e0217102. doi: 10.1371/journal.pone.0217102 (PMC6581242; doi:10.1371/journal.pone.0217102)
Supplement: S1 File — (ZIP) [file pone.0217102.s001.zip › Qualitative Data Collection tool/INTERVIEW GUIDE - PERINATAL WOMEN ENGLISH.docx]

**PERINATAL DEPRESSION STUDY**

**Open ended interview guide – For in-depth interviews with perinatal women (English)**

**Section A: Demographic details**

1. Age:
2. Marital status: Probe; if married is this a monogamous or polygamous relationship?

How many wives are they? Which number are they?

Also probe how many times they have been married

If divorced, probe on issues that led to the divorce

1. Parity: Probe if the current pregnancy was planned, if not how they feel about it.
2. Number of live children:

Probe: causes of death of the child/ children if any, Date of deaths

And how the death of their child/children affects them

1. Religion:
2. HIV Status

Probe: how their HIV status affects them

1. Residential address: Type of accommodation? Number of people living in the house?
2. Occupation:

Probe: who is a bread winner in the family?

If not working, how do they spend their time/day?

**Section B: Questions related to perinatal depression**

We are concerned that you may be struggling with depression or feeling “not yourself”

1. What does depression mean to you?

Probes: tell me more about what you have been feeling? Your mood, eating and sleeping

pattern, energy level, interest or pleasure in things, thoughts of self-harm

Were you aware that you have this problem?

How did you find out you had this problem? Did someone in your family/community or a health care provider express concern?

How does it affect your functioning - has depression made it hard for you to do your work, take care of your baby, take care of things at home, or get along with other people?

1. What do you think has caused this problem?
2. Tell me about your childhood experiences

Probes: How they grew up, any challenges, relationships problems, any history of physical and or mental health problems

1. Tell me more about your experiences during this pregnancy/ delivery and postpartum

Probes: Challenges during pregnancy/ delivery and postpartum

Family experiences/family life

Relationship with husband /spouse and significant others (further probe support from spouse/husband; material/emotional support, how much)

1. Tell me about any experience of trauma, neglect or violence you have experiences; from spouse or from someone else in your life?

How do these experiences affect you?

1. Can you tell me what you have been doing to cope with your challenges?

Probes: seeking help - from spouse/family/friends; traditional and spiritual leaders

Probe on Kind of help

Self-help initiatives; Probe on types of self-initiatives

1. If you could have one thing to support you, what would it be?

Probes: From spouse, family, work, friends and community, from health centre

1. What assistance do you think health care providers can offer you?

Probe: Would you take part in counselling at a health facility?

Would you take part in counselling or any form of talk therapy in your community?

What type of a person/people do you think would be appropriate to offer such kind of help?

Would you be willing to take medication to treat the depression?

Would you have concerns to be involved in counselling and/or taking medication?

If YES, what are your concerns?

How would your family react to your seeking help or participating in counselling?
