# Supplementary material for: “Passing through difficult times”: Perceptions of perinatal depression and treatment needs in Malawi - A qualitative study to inform the development of a culturally sensitive intervention
Source: PLoS One. 2019 Jun 18;14(6):e0217102. doi: 10.1371/journal.pone.0217102 (PMC6581242; doi:10.1371/journal.pone.0217102)
Supplement: S1 File — (ZIP) [file pone.0217102.s001.zip › Qualitative Data Collection tool/Key Informant Interview Guide with primary health care workers and maternal health coordinators.docx]

**PERINATAL DEPRESSION STUDY**

**Key Informant Interview Guide (PHC workers and maternal health coordinators)**

**Section A: Demographic details**

1. Age:
2. Professional qualification:
3. Position:
4. Department:

Responsibilities

Catchment area/Jurisdiction

1. Address:
2. Years of service:

**Section B: Questions related to perinatal depression**

1. What is your understanding of perinatal depression?

**Probes:** Its causes, how common is the problem in your catchment area?

Have they encountered any women with depression in the perinatal period? How do they identify women who have perinatal depression? What do these women present with?

How do they manage women with perinatal depression?

1. How are your views regarding depression in pregnancy or postpartum period?

**Probe:** A health care issue, Do the community or policy makers realise that perinatal depression is a health care issue?

1. Do you know any complications of perinatal depression?

**Probes:** to the sufferer

to the unborn child/infant/child

to the family

1. Are there any efforts made within safe motherhood initiative/Reproductive Health to address this problem

**Probes:** what is being done, by who?

1. In your view what interventions do you think can best help women with perinatal depression?

**Probes:** Pharmacological: drugs they can use

Desirability of medication in perinatal period

Nonpharmacological: Education interventions, counselling, Individual Vs group interventions

For nonpharmacological

**Probes**: have these interventions been implemented at this facility?

Were you involved?

How effective do you think these interventions are?

1. Can such interventions be implemented in primary health care setting and the community?

Probes: Who/which cadre would best implement the intervention?

Specialists, PHC workers, Lay volunteers

Probe: why they chose a specific cadre

Skills to providing the intervention

Acceptability and feasibility of the interventions

1. What would hinder provision of interventions for perinatal depression? Be it pharmacological or nonpharmacological.
